# Temperament Type Specific Metabolite Profiles of the Prefrontal Cortex and Serum in Cattle

**DOI:** 10.1371/journal.pone.0125044

**Published:** 2015-04-30

**Authors:** Bodo Brand, Frieder Hadlich, Bettina Brandt, Nicolas Schauer, Katharina L. Graunke, Jan Langbein, Dirk Repsilber, Siriluk Ponsuksili, Manfred Schwerin

**Affiliations:** 1 Leibniz Institute for Farm Animal Biology, Institute of Genome Biology, Dummerstorf, Germany; 2 Metabolomic Discoveries GmbH, Golm, Germany; 3 Leibniz Institute for Farm Animal Biology, Institute of Behavioral Physiology, Dummerstorf, Germany; 4 University of Rostock, Institute for Farm Animal Research and Technology, Rostock, Germany; 5 Leibniz Institute for Farm Animal Biology, Institute of Genetics and Biometry, Dummerstorf, Germany; Technion - Israel Institute of Technology, ISRAEL

## Abstract

In the past decade the number of studies investigating temperament in farm animals has increased greatly because temperament has been shown not only to affect handling but also reproduction, health and economically important production traits. However, molecular pathways underlying temperament and molecular pathways linking temperament to production traits, health and reproduction have yet to be studied in full detail. Here we report the results of metabolite profiling of the prefrontal cortex and serum of cattle with distinct temperament types that were performed to further explore their molecular divergence in the response to the slaughter procedure and to identify new targets for further research of cattle temperament. By performing an untargeted comprehensive metabolite profiling, 627 and 1097 metabolite features comprising 235 and 328 metabolites could be detected in the prefrontal cortex and serum, respectively. In total, 54 prefrontal cortex and 51 serum metabolite features were indicated to have a high relevance in the classification of temperament types by a sparse partial least square discriminant analysis. A clear discrimination between fearful/neophobic-alert, interested-stressed, subdued/uninterested-calm and outgoing/neophilic-alert temperament types could be observed based on the abundance of the identified relevant prefrontal cortex and serum metabolites. Metabolites with high relevance in the classification of temperament types revealed that the main differences between temperament types in the response to the slaughter procedure were related to the abundance of glycerophospholipids, fatty acyls and sterol lipids. Differences in the abundance of metabolites related to C21 steroid metabolism and oxidative stress indicated that the differences in the metabolite profiles of the four extreme temperament types could be the result of a temperament type specific regulation of molecular pathways that are known to be involved in the stress and fear response.

## Introduction

Temperament in cattle can be defined as the consistent behavioral and physiological difference observed between individuals in response to a stressor or environmental challenge and is used to describe the relatively stable difference in the behavioral predisposition of an animal, which can be related to psychobiological mechanisms [1–3]. Cattle temperament has been shown to be related to the variation of several economically important production traits like carcass and meat quality in beef cattle [4] or milk yield in dairy cattle [2,5], as well as to have an impact on health [6,7] and reproduction. Additionally, a link between cattle temperament and stress responsiveness as well as between temperament and fear responsiveness has been suggested [8–10]. In dairy cattle for example, Sutherland et al. [2] showed, that animals that need less time (less than 2 s) to exit a squeeze chute had a higher baseline concentration of cortisol compared to cattle that had exit times of more than 4 s. The animals that had lower exit times had also lower milk yields when milked in a novel environment [2], overall indicating that animals with more excitable temperaments seem to have higher baseline cortisol levels and are more prone to losses in productivity. Studies in mice and rat have shown that the selection for a specific behavior phenotype, like the learned helplessness model of depression in rats [11,12] or the high and low anxiety-related behavior model in mice [13,14], is possible, and several studies in cattle have identified quantitative trait loci for behavior related and temperament related traits [15–19] overall indicating a genetic background of behavior. Therefore, it is assumed that the selection for temperament types that are well suited for specific production systems will improve productivity and overall animal welfare [6,20,21].

One of the main difficulties in studying cattle temperament is the complexity and the assessment of temperament itself [22]. Generally cattle temperament is assumed to be multidimensional and Réale et al. [22], for example, proposed five underlying categories of temperament traits: shyness-boldness, exploration-avoidance, activity, aggressiveness and sociability. Fear is considered as one of the main psychological factors underlying temperament traits [20,23] and several behavioral tests have been developed or have been adapted from other species to measure temperament traits in cattle [22,24]. Due to the complexity of behavioral traits and because behavioral tests are specific in regard to the temperament traits that can be recorded [22], there is no single objective measurement that is able to capture all characteristics of temperament [20]. Therefore, several studies have also included physiological and endocrinological measurements to evaluate, for example, the activity of the hypothalamic-pituitary-adrenal axis (e.g. cortisol) and sympatho-adrenal medullary system (e.g. epinephrine, heart rate) which are well known systems in the stress response.

To gain further insights into molecular pathways affecting temperament in cattle and to identify metabolites that could improve the assessment of temperament, we used gas chromatography (GC) and liquid chromatography (LC) coupled to mass spectrometry (MS) to detect known and unknown metabolites in the prefrontal cortex and serum of cows with differing temperament types and show that a discrimination of distinct temperament types is possible based on the abundance of prefrontal cortex and serum metabolite features.

## Material and Methods

### Animals, housing and slaughter conditions

Twenty-five cows deriving from a F2 resource population established from a cross between Charolais and German Holstein founder breeds (SEGFAM resource population, [25]) were investigated in this study. The animals were selected from a total of 184 cows for distinct differences in their temperament assessed in a novel object and novel human test 90 days post natum [26]. All animals were housed in a loose housing barn under identical environmental and feeding conditions at the Leibniz Institute for Farm Animal Biology (FBN) in Dummerstorf, Germany [27]. The animals were slaughtered at day 30 after parturition in their second lactation at an age of 1341 ± 98 days. At the day of slaughter the cows were milked between 4:00 a.m. and 6:30 a.m.. Fifteen to thirty minutes before slaughter the animals were transported to the European Union accredited slaughterhouse of the FBN, which is in close vicinity to the barn (about 500m). The animals were killed during the regular slaughter process between 7:45 a.m. and 9:45 a.m.. A captive bolt pistol was used for stunning and the animals were killed by bleeding. The experimental procedures were carried out according to the animal care guidelines of the State Mecklenburg-Vorpommern, Germany, and were approved by the Landesamt für Landwirtschaft, Lebensmittelsicherheit und Fischerei Mecklenburg-Vorpommern (Reference number: LVL M-V/310-4/7221.3–2.1-017/03).

### Behavioral Classification and Animal Selection

At the age of 90 days all SEGFAM calves were subjected to a novel object test and a novel human test, in a 9.6 m x 4.0 m open field that was divided in four segments of 2.4 m length. A detailed description of the experimental setup, the recorded behaviors and the analyzes performed to assess the temperament types is given in Graunke et al. [26] for the novel object test. The novel human test was performed in accordance to the novel object test subsequent to the novel object test by exchanging the novel object (traffic pylon) with a staff person unknown to the calf. Briefly, measurements of behaviors were live-recorded during the two tests that lasted ten minutes each using the observation software tool The Observer 5.0 (Noldus, The Netherlands). Recorded behaviors were: contact with the novel object or human; inactivity; exploration; grooming; activity; running; vocalization; changes between segments; the habituation of the calf in the open field segment harboring the novel object or human and the habituation of the calf in the neighboring segment. The data were analyzed using a principal component analysis (PCA). The first two principal components (PC) explained 46.8% and 11.2% of the variance in the novel object [26] and 45.0% and 16.9% in the novel human test, respectively. In both tests the first two PCs were predominantly influenced by behaviors comprising contact to the novel object or human and the time spend near the object or human (PC1), as well as by the exploration of the open field and the inactivity of the calves during the tests (PC2) [26]. Highest loadings in PC1 were observed for the duration (0.764 and 0.947), frequency (0.832 and 0.947) and latency (-0.896 and -0.942) of contact with the novel object or human and latency (-0.872 and -0.88) and duration (0.838 and 0.91) of the time spend near the object or human, respectively. In PC2 the highest loadings were observed for the duration of inactivity (0.855 and 0.897) and exploration of the open field (0.83 and 0.80) in the novel object and novel human test, respectively (S1 File). Based on the PC-scores of PC1 and PC2 that were calculated for each calf and each test the calves were assigned into five groups. In regard to the exploration and avoidance of the novel object or human (PC1), the inactivity and exploration of the open field (PC2) and the heart rate variability, four extreme phenotypes were identified that were described as “fearful/neophobic-alert” (low PC1-scores and high PC2-scores), “interested-stressed” (high PC1- and PC2-scores), “outgoing/neophilic-alert” (high PC1-scores and low PC2-scores) and “subdued/uninterested-calm” (low PC1- and PC2-scores) temperament types, respectively [26]. The animals in the fifth group showed no distinct response and were described as indistinct. For each distinct temperament type five animals that showed an extreme response at least in one of the tests and the most consistent behavioral response to the other test were selected from all 184 available SEGFAM cows. For the indistinct group five animals were selected that showed no distinct response in both tests.

### Sample Preparation

Blood samples were taken from the jugular vein while the animals were restrained in a standing stock within the slaughterhouse right before the animals were stunned and slaughtered. The blood was collected in serum-tubes (S-Monovette, Sarstedt, Nümbrecht, Germany). Within 30 min the samples were transferred to the laboratory. After centrifugation the serum samples were prepared, aliquoted and immediately stored at -80°C until they were sent to Metabolomic Discoveries GmbH on dry ice for analysis. Average processing time for the blood-samples was about 70 min.

Prefrontal cortex samples were immediately taken after the cows were killed by bleeding and the head was removed. A 0.5-cm slice was taken from the right anterior prefrontal cortex region by a coronal section. The whole slice was cut in small pieces and immediately frozen in liquid nitrogen and stored at -80°C until the samples were sent to Metabolomic Discoveries GmbH on dry ice for analysis. Average processing time for prefrontal cortex samples was between 30 and 45 min after stunning. All brain samples were prepared by a single person for consistency.

### Metabolite profiling

All subsequent steps were carried out at Metabolomic Discoveries GmbH (Potsdam, Germany; www.metabolomicdiscoveries.com). Frozen prefrontal cortex tissue was mechanically disrupted in a ball mill in liquid nitrogen. 60 mg of homogenate was mixed with 1 ml 80% (v/v) methanol and incubated for 15 min in a thermoshaker (1000 rpm) at 70°C. Cellular debris was removed by centrifugation for 15 min at 17136 rcf. Blood serum was mixed with nine volumes of 90% (v/v) methanol and incubated for 15 min at 37°C with vigorous shaking. Precipitated proteins were separated from the extract by centrifugation for 15 min at 17136 rcf. Metabolite extracts were analyzed in parallel on a GC-MS and UPLC-QTOF/MS. Derivatisation and analyses of metabolites by a GC-MS 7890A mass spectrometer (Agilent, Santa Clara, USA) were carried out as described elsewhere [28]. For GC-MS, metabolites were identified in comparison to Metabolomic Discoveries' database entries of authentic standards. The LC separation was performed using Zorbax SB-Aq column (Agilent, Santa Clara, USA), operated by an Agilent 1290 UPLC system (Agilent, Santa Clara, USA). The LC mobile phase was A) 0.1% (v/v) formic acid in water and B) 0.1% (v/v) formic acid in methanol with a gradient from 0% B to 90% over 5 min, to 95% at 6.5 min and 100% at 8 min, subsequently equilibrate. The flow rate was 400 μl/min, injection volume 1 μl. Mass spectrometry was performed using a high-resolution 6540 QTOF/MS Detector (Agilent, Santa Clara, USA) with a mass accuracy of < 2ppm. For UPLC-QTOF/MS, metabolites were identified or putatively annotated in comparison to Metabolomic Discoveries' database entries of authentic standards and METLIN database entries [29] through peak mass within 5ppm mass accuracy and retention time. Within the text no discrimination between identified (GC-MS and UPLC-QTOF/MS) and putatively annotated metabolites (UPLC-QTOF/MS) is made and both are referred to as metabolites. The measured metabolite concentration (GC-MS and UPLC-QTOF/MS) was normalized to internal standards and the fresh weight of the sample. Outlier samples within the metabolite profiles were detected by performing a PCA with JMP Genomics 5.1 (SAS Institute Inc., Cary, NC, 1989–2007) for the prefrontal cortex and serum metabolite data separately. The serum sample of one interested-stressed animal had to be excluded from further analysis because the sample was an extreme outlier in the PCA indicating issues related to the preparation of the sample. PC-scores of the first principal component were in the range of -13.2 to +13.8 for all animals except for the outlier that had a PC-score of 64.4. In addition, one outgoing/neophilic-alert animal was removed from the study because it was suspicious in the PCA and further inquiries revealed health problems. After quality control 24 prefrontal cortex and 23 serum samples were considered for all subsequent analyses. The final dataset consisted of a list of metabolite features comprising identified (GC-MS), putatively annotated (UPLC-QTOF/MS) and unknown metabolite features as well as the technique used to detect them and their relative abundance.

### Statistical Analysis

To characterize the complete prefrontal cortex and serum metabolite data a PCA with JMP Genomics 5.1 (SAS Institute Inc., Cary, NC, 1989–2007) of all prefrontal cortex and serum metabolite features was performed for each tissue separately, and identified and putatively annotated metabolites were mapped to LIPID MAPS- [30], HMDB- [31], KEGG- [32,33] or PubChem-Compound identifiers (CID) [34] for the functional characterization of the metabolites using Ingenuity pathway analysis (IPA^®^, QIAGEN Redwood City, www.quiagen.com ingenuity).

To explore the prefrontal cortex and serum metabolite data and identify metabolite features with a high relevance in the classification of temperament types the statistical learning method sparse partial least squares discriminant analysis (sPLS-DA; [35]) was applied. The analyses were realized in R [36] using the R package mixOmics version 4.1.5 [37]. Datasets were log transformed, mean-centered and unit variance scaled. For all analyses the response matrix *Y* (temperament types) was recoded as a dummy block matrix by recoding the temperament type of each animal using a dummy variable. To select the optimal combination of kept variables/metabolites (*nkeep*) and components (*ncomp*) that are needed as input parameters in the sparse analyses and to identify metabolites that have a high relevance in the classification of all temperament types, an exploratory approach comprising two nested leave-one-out-cross-validations [38] was applied. The analyses were performed for *nkeep* ranging from 1 to 100 and *ncomp* ranging from 1 to 10 and the most robust combination of *nkeep* and *ncomp* that was used as input parameters for the classification of temperament types was identified by minimizing the mean squared error of prediction (msep).

Briefly, in the first step of the cross-validation design, a leave-one-out-cross-validation, termed outer cross validation was performed to exclude metabolite features unimportant in the classification of temperament types. For each outer cross validation run one sample was chosen as outer test set and all remaining samples were defined as outer training set. The variable important in the projection (VIP) coefficients [39] were calculated for each combination of *nkeep* and *ncomp* using the outer training sets and metabolites and metabolite features with VIP coefficients unequal to zero were used in the second step of the cross validation design, termed inner cross validation. The inner cross-validation was a second leave-one-out cross-validation that was performed to identify the optimal number of components used for the classification. For each inner cross validation run one of all outer training set samples was chosen as inner test set and all remaining outer training set samples were defined as inner training set. The msep across all inner cross validation runs was calculated for each combination of *nkeep* and *ncomp*, and the optimal number of components was identified by the lowest msep. Finally, in the third step of the cross validation design, the optimal combination of *nkeep* and *ncomp* and the metabolites with a high relevance in the classification of temperament types were identified using the outer test sets. Therefore, the msep for each combination of *nkeep* and *ncomp* was calculated based on the VIP-variables selected in the first step and the optimal *ncomp* identified in the second step of the cross-validation design. All mseps were ranked from the lowest to the highest values and recorded in a matrix with dimension *nkeep* × *ncomp* to calculate a smoothed rank score by applying a 3 × 3 convolution matrix (1).

$$\left[\begin{array}{ccc} 0 & 1 & 0\\ 1 & 2 & 1\\ 0 & 1 & 0 \end{array}\right]$$ (1)

The combination of *nkeep* and *ncomp* with the lowest smoothed rank score was assumed to be the optimal and robust combination of *nkeep* and *ncomp* to be used for the classification and all metabolites with a mean VIP-score across any outer cross-validation run higher or equal to one were expected to have a high relevance in the classification of temperament types. Additionally, the PCA and the Kruskal-Wallis-Test were used to visualize and confirm the results of the sPLS-DA and the Wilcoxon-Mann-Whitney-Test was applied to identify nominal significant differences in the abundance of metabolites between temperament types. To visualize the differences in the abundance of metabolites a two-way hierarchical clustering was performed and a heatmap was drawn.

## Results

### Metabolite Profiling

In total, 627 and 1097 metabolite features were detected by GC-MS and UPLC-QTOF/MS analysis of the prefrontal cortex (Table A in S2 File) and serum samples (Table B in S2 File), respectively. For 235 and 328 of the features, the corresponding metabolites could be identified or putatively annotated and 126 and 149 of these metabolites could be mapped to LIPID MAPS- [30], HMDB- [31], KEGG- [32,33] or PubChem-Compound-identifiers [34], respectively. 81 of all metabolite features were detected in both the prefrontal cortex and serum samples. All other metabolite features were identified only in one of the two tissues.

**Fig 1:**
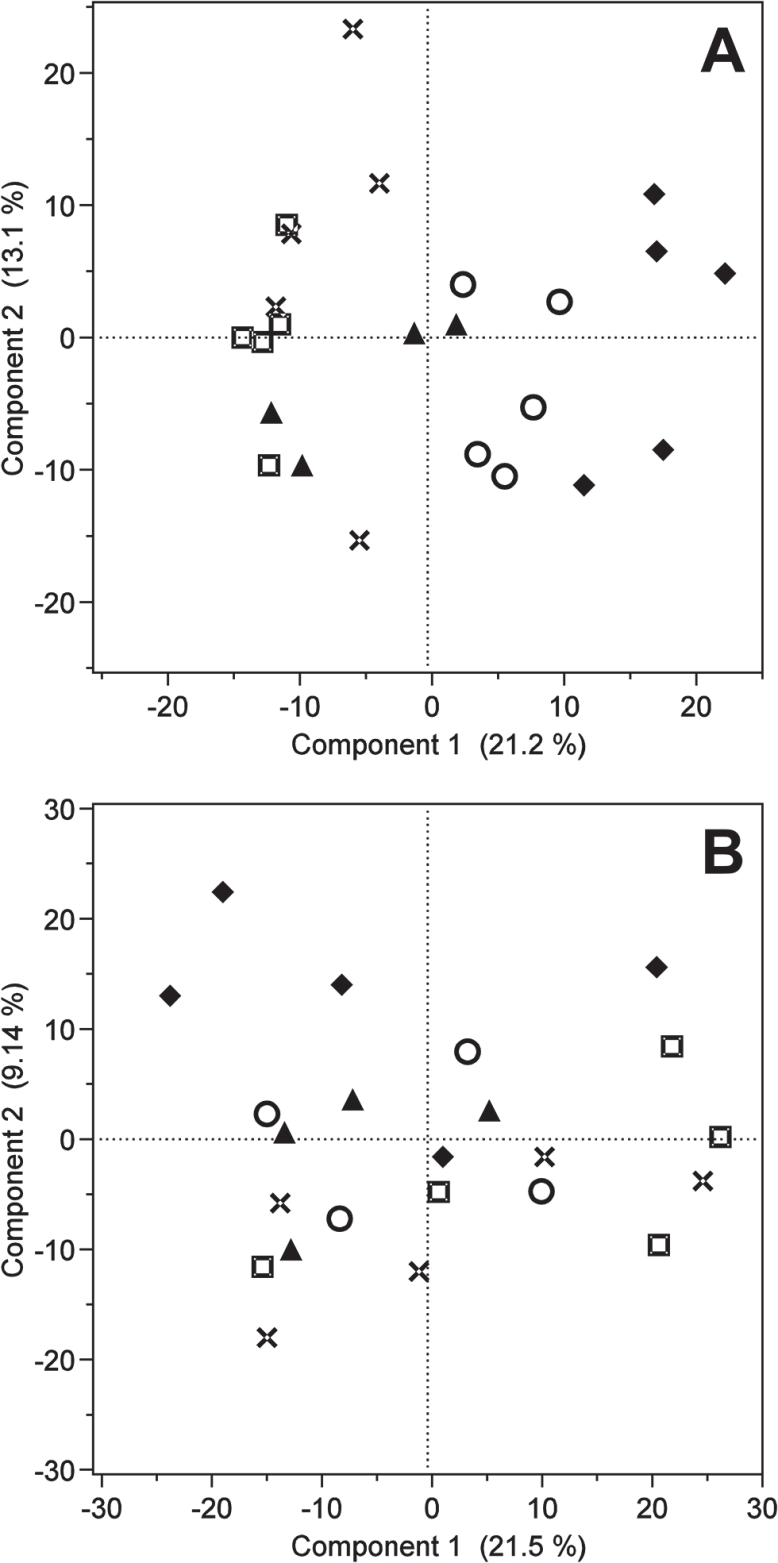
Principal component score plot for prefrontal cortex and serum metabolites. Principal component score plot of the first two principal components for (A) the complete prefrontal cortex data and (B) the complete serum data. Symbols indicate the different temperament types, rhombs the fearful/neophobic-alert, circles the interested-stressed, triangles the outgoing/neophilic-alert, squares the subdued/uninterested-calm and crosses the indistinct temperament type.

Ingenuity pathway analysis (IPA^®^, QIAGEN Redwood City, www.quiagen.com ingenuity) of mapped prefrontal cortex and serum metabolites indicated that most of the identified and putatively annotated metabolites are involved in the molecular and cellular functions comprising amino acid metabolism, molecular transport and small molecule biochemistry. PCA of the complete prefrontal cortex metabolite data and plotting of the PC-scores for the first two PCs indicated temperament type specific difference based on the abundance of prefrontal cortex metabolite features at least for the distinct temperament types (Fig 1A), whereas no obvious differentiation could be observed for the serum data (Fig 1B).

### Metabolites with high relevance in the classification of temperament types

**Fig 2:**
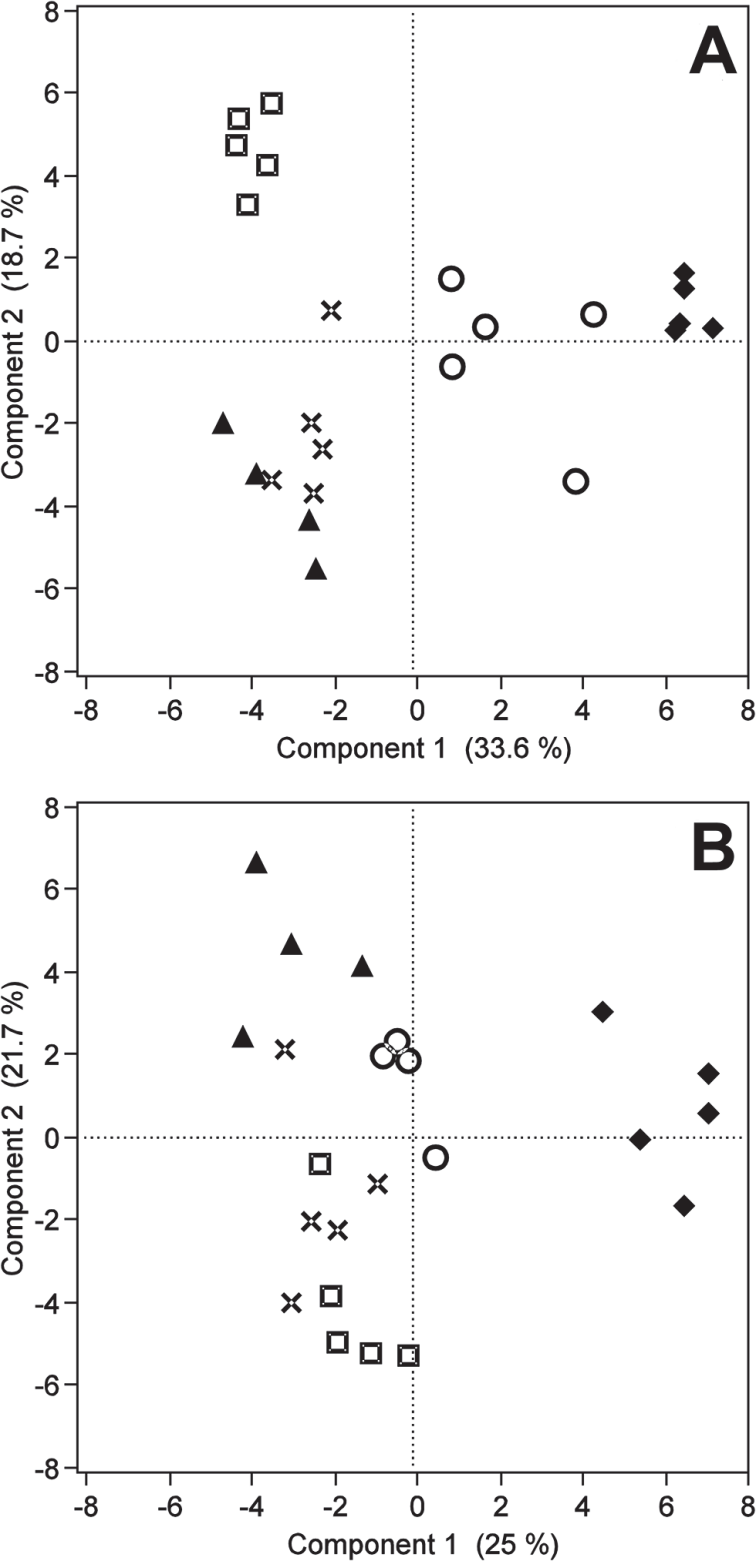
Principal component score plot for metabolites with high relevance. Principal component score plot of the first two principal components for metabolite features with high relevance in the classification of temperament types in the prefrontal cortex (A) and serum (B). Symbols indicate the different temperament types, rhombs the fearful/neophobic-alert, circles the interested-stressed, triangles the outgoing/neophilic-alert, squares the subdued/uninterested-calm and crosses the indistinct temperament type.

**Fig 3:**
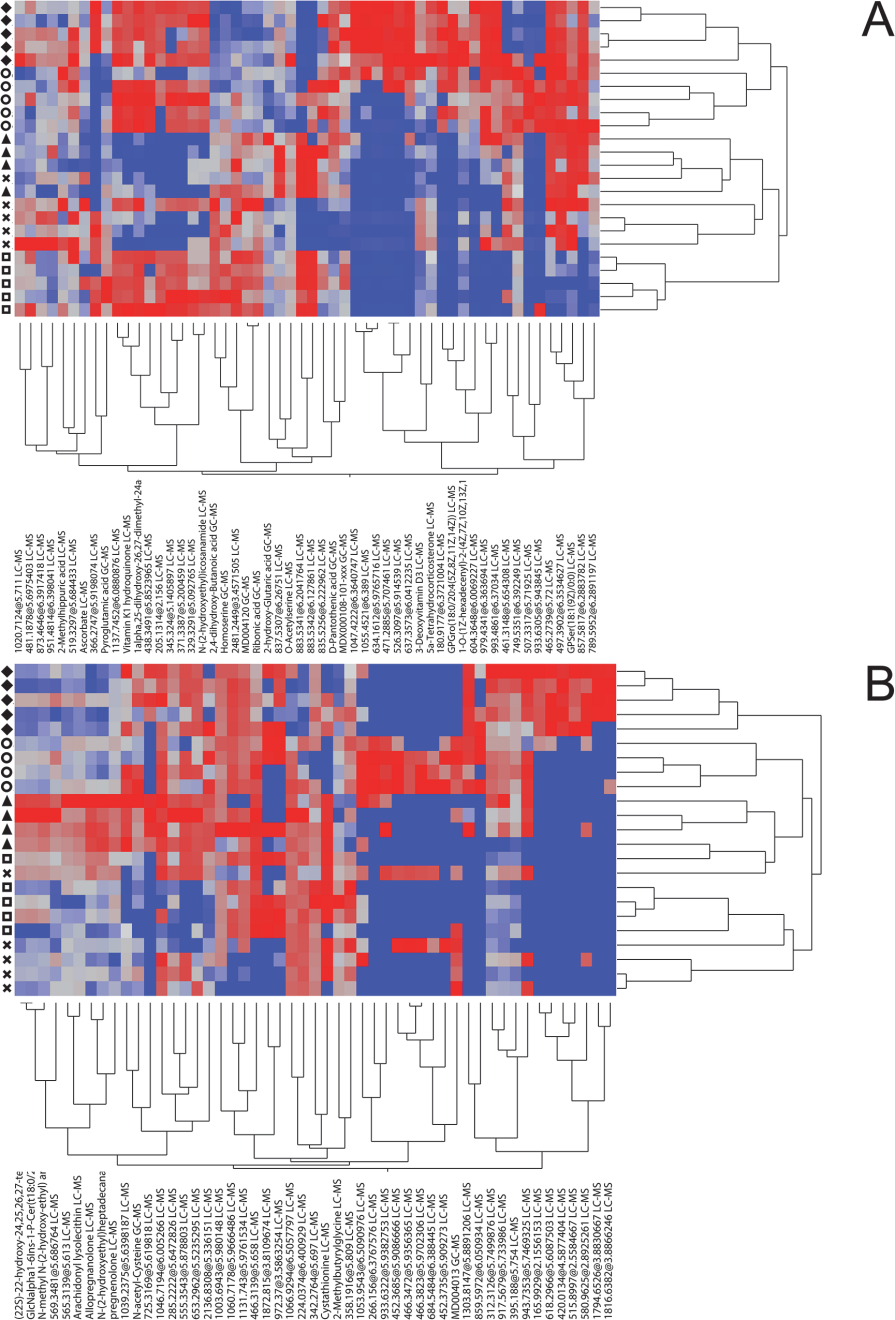
Hierarchical clustering of metabolites with high relevance. Two way hierarchical clustering of (A) prefrontal cortex and (B) serum metabolite features with a high relevance in the classification of temperament types. Symbols indicate the different temperament types, rhombs the fearful/neophobic-alert, circles the interested-stressed, triangles the outgoing/neophilic-alert, squares the subdued/uninterested-calm and crosses the indistinct temperament type.

Metabolites that had a high relevance in the classification of temperament types were identified by a sPLS-DA applying a nested cross-validation design. The optimal combination of components and variables to be used in the sPLS-DA classification models were 4 components with 23 variables for the prefrontal cortex data and 4 components with 14 variables for the serum data. By applying the VIP-algorithm 54 prefrontal cortex and 51 serum metabolite features were identified to have a high relevance in the classification of temperament types (VIP-score > 1) (Table A and Table B in S3 File). Metabolites with high relevance are shown in Table 1. The msep across all temperament types was 0.074 for the prefrontal cortex data and 0.097 for the serum data. The sPLS-DA classification models were able to explain 89% of the variance between temperament types for the prefrontal cortex data and about 83% of the variance for the serum data, respectively. PCA of the 54 prefrontal cortex (Fig 2A) and 51 serum metabolite features (Fig 2B) selected in the sPLS-DA and plotting of the PC-scores for the first two PCs showed that each distinct temperament type built a separate cluster discriminating temperament types from each other. In addition, the analysis also indicated tissue specific differences in the discrimination of temperament types. In the prefrontal cortex, the largest differences in terms of numbers of differentially regulated metabolites were observed between the fearful/neophobic-alert and the subdued/uninterested-calm temperament type and less differences between the outgoing/neophilic-alert and indistinct animals. In serum the largest differences were observed between the fearful/neophobic-alert and all other temperament types and the indistinct cows were spread in between the interested-stressed, subdued/uninterested-calm and outgoing/neophilic-alert temperament types (Fig 2B). This can also be observed in the hierarchical clustering of the most relevant metabolites (Fig 3), especially in the serum, where the fearful/neophobic-alert animals built a separate cluster (Fig 3B). The differences in the prefrontal cortex are mainly attributed to the high abundance of metabolite features and metabolites like the sterol lipids 3-Deoxyvitamin D3 and 5a-Tetrahydrocorticosterone or glycerophospholipids PE(P-16:0/22:6) and GPGro(18:0/20:4) in the fearful/neophobic-alert animals (Fig 3A). In serum the differences are mainly attributed to the high abundance of unknown metabolite features in the fearful/neophobic-alert animals and the high abundance of metabolite features and metabolites like pregnenolone and allopregnanolone in the outgoing/neophilic-alert animals (Fig 3B). A detailed list of the metabolite features with high relevance in the classification of temperament types, comprising mean VIP-scores, amount of occurrences in the cross-validation runs as well as the p-values of the Kruskal-Wallis-Test are given in Table 1 for the prefrontal cortex and serum metabolites with high relevance and in supplemental S3 File for all prefrontal cortex (Table A in S3 File) and serum (Table B in S3 File) metabolite features with high relevance including the results of the Kruskal-Wallis-Test and the Wilcoxon-Mann-Whitney-Test. 48 of the prefrontal cortex metabolite features and 42 of the serum metabolite features with high relevance also showed a nominal significance in the Kruskal-Wallis-Test (p ≤ 0.05), and 51 and 49 metabolite features showed a nominal significant difference in at least one comparison between temperament types in the Wilcoxon-Mann-Whitney-Test (p ≤ 0.05), respectively. Metabolites with high relevance and their classification based on the LIPID MAP classification system [30] for lipids are given in Table 2 for the prefrontal cortex and in Table 3 for serum metabolites, if available. In total, 17 lipids and 10 other metabolites were identified to have a high relevance in the classification of temperament types. The results of the Kruskal-Wallis-Test and Wilcoxon-Mann-Whitney-Test for the complete metabolite data are provided in supplemental S2 File for all prefrontal cortex (Table A in S2 File) and serum (Table B in S2 File) metabolite features.

**Table 1:** Metabolites with high relevance in the classification of temperament types.

Metabolite Feature Name	Technique	Tissue	VIP	Occurrence Cross-validation	Kruskal-Wallis-Test
2,4-dihydroxy-Butanoic acid	GC-MS	PC	6.51	24	0.0006
N-(2-hydroxyethyl)heptadecanamide	LC-MS	S	4.31	17	0.0187
GPSer(18:1(9Z)/0:0)	LC-MS	PC	4.05	24	0.0048
Ribonic acid	GC-MS	PC	3.85	24	0.0028
1alpha,25-dihydroxy-26,27-dimethyl-24a-homovitamin D3 / 1alpha,25-dihydroxy-26,27-dimethyl-24a-homocalciferol	LC-MS	PC	3.81	24	0.0088
N-acetyl-Cysteine	GC-MS	S	3.79	16	0.0230
Arachidonyl lysolecithin	LC-MS	S	3.27	17	0.0212
N-methyl N-(2-hydroxy-ethyl) arachidonoyl amine	LC-MS	S	3.16	16	0.0316
Allopregnanolone	LC-MS	S	2.91	15	0.0183
O-Acetylserine	LC-MS	PC	2.75	23	0.0107
Vitamin K1 hydroquinone	LC-MS	PC	2.68	23	0.0059
(22S)-22-hydroxy-24,25,26,27-tetranorvitamin D3 / (22S)-22-hydroxy-23,24,25,26,27-pentanorcholecalciferol	LC-MS	S	2.55	16	0.0257
GPGro(18:0/20:4(5Z,8Z,11Z,14Z)) / GPGro(18:0/20:4)	LC-MS	PC	2.47	24	0.0014
1-O-(1Z-hexadecenyl)-2-(4Z,7Z,10Z,13Z,16Z,19Z-docosahexaenoyl)-sn-glycero-3-phosphoethanolamine / PE(P-16:0/22:6	LC-MS	PC	2.03	24	0.0026
5a-Tetrahydrocorticosterone	LC-MS	PC	1.98	16	0.0056
Cystathionine	LC-MS	S	1.86	17	0.0216
3-Deoxyvitamin D3	LC-MS	PC	1.75	23	0.0078
D-Pantothenic acid	GC-MS	PC	1.70	21	0.0020
Ascorbate	LC-MS	PC	1.70	23	0.0136
GlcNalpha1-6Ins-1-P-Cer(t18:0/26:0)	LC-MS	S	1.68	12	0.0221
Pyroglutamic acid	GC-MS	PC	1.60	23	0.0410
Homoserine	GC-MS	PC	1.43	20	0.0046
2-Methylbutyrylglycine	LC-MS	S	1.40	7	0.0210
N-(2-hydroxyethyl)icosanamide	LC-MS	PC	1.35	21	0.0049
Pregnenolone	LC-MS	S	1.22	6	0.0285
2-hydroxy-Glutaric acid	GC-MS	PC	1.18	21	0.2351

Metabolite names, measuring technique and their VIP-Scores, number of occurrences in the outer cross-validation runs and nominal *p*-values for the Kruskal-Wallis-Test are shown for serum (S) and prefrontal cortex (PC) metabolites with high relevance in the classification of temperament types.

**Table 2:** Annotation and classification of prefrontal cortex metabolites with high relevance in the classification of temperament types.

Metabolite Feature Name	Lipid Maps ID	Category	Main Class	Sub Class
2,4-dihydroxy-Butanoic acid	LMFA01050385	Fatty Acyls [FA]	Fatty Acids and Conjugates [FA01]	Hydroxy fatty acids [FA0105]
Ribonic acid	LMFA01050411	Fatty Acyls [FA]	Fatty Acids and Conjugates [FA01]	Hydroxy fatty acids [FA0105]
N-(2-hydroxyethyl)icosanamide	LMFA08040038	Fatty Acyls [FA]	Fatty amides [FA08]	N-acyl ethanolamines (endocannabinoids) [FA0804]
1-O-(1Z-hexadecenyl)-2-(4Z,7Z,10Z,13Z,16Z,19Z-docosahexaenoyl)-sn-glycero-3-phosphoethanolamine / PE(P-16:0/22:6)	LMGP02030001	Glycerophospholipids [GP]	Glycerophosphoethanolamines [GP02]	1-(1Z-alkenyl),2-acylglycerophosphoethanolamines [GP0203]
GPSer(18:1(9Z)/0:0)	LMGP03050001	Glycerophospholipids [GP]	Glycerophosphoserines [GP03]	Monoacylglycerophosphoserines [GP0305]
GPGro(18:0/20:4(5Z,8Z,11Z,14Z)) / GPGro(18:0/20:4)	LMGP04010039	Glycerophospholipids [GP]	Glycerophosphoglycerols [GP04]	Diacylglycerophosphoglycerols [GP0401]
Vitamin K1 hydroquinone	LMPR02030030	Prenol Lipids [PR]	Quinones and hydroquinones [PR02]	Vitamin K [PR0203]
5a-Tetrahydrocorticosterone	LMST02030142	Sterol Lipids [ST]	Steroids [ST02]	C21 steroids (gluco/mineralocorticoids, progestogins) and derivatives [ST0203]
1alpha,25-dihydroxy-26,27-dimethyl-24a-homovitamin D3 / 1alpha,25-dihydroxy-26,27-dimethyl-24a-homocalciferol	LMST03020464	Sterol Lipids [ST]	Secosteroids [ST03]	Vitamin D3 and derivatives [ST0302]
3-Deoxyvitamin D3	LMST03020618	Sterol Lipids [ST]	Secosteroids [ST03]	Vitamin D3 and derivatives [ST0302]
**Metabolite Feature Name**	**HMDB**	**Kegg Compund ID**		
Ascorbate	HMDB00044	C00072		
D-Pantothenic acid	HMDB00210	C00864		
Pyroglutamic acid	HMDB00267	C01879		
2-hydroxy-Glutaric acid	HMDB00694	C03196		
Homoserine	HMDB00719	C00263		
O-Acetylserine	HMDB03011	C00979		
2-Methylhippuric acid	HMDB11723			

**Table 3:** Annotation and classification of serum metabolites with high relevance in the classification of temperament types.

Metabolite Feature Name	Lipid Maps ID	Category	Main Class	Sub Class
N-methyl N-(2-hydroxy-ethyl) arachidonoyl amine	LMFA08020025	Fatty Acyls [FA]	Fatty amides [FA08]	N-acyl amines [FA0802]
N-(2-hydroxyethyl)heptadecanamide	LMFA08040049	Fatty Acyls [FA]	Fatty amides [FA08]	N-acyl ethanolamines (endocannabinoids) [FA0804]
Arachidonyl lysolecithin	LMGP01050048	Glycerophospholipids [GP]	Glycerophosphocholines [GP01]	Monoacylglycerophosphocholines [GP0105]
GlcNalpha1-6Ins-1-P-Cer(t18:0/26:0)	LMSP06040001	Sphingolipids [SP]	Acidic glycosphingolipids [SP06]	Phosphoglycosphingolipids [SP0604]
pregnenolone	LMST02030088	Sterol Lipids [ST]	Steroids [ST02]	C21 steroids (gluco/mineralocorticoids, progestogins) and derivatives [ST0203]
Allopregnanolone	LMST02030130	Sterol Lipids [ST]	Steroids [ST02]	C21 steroids (gluco/mineralocorticoids, progestogins) and derivatives [ST0203]
(22S)-22-hydroxy-24,25,26,27-tetranorvitamin D3 / (22S)-22-hydroxy-23,24,25,26,27-pentanorcholecalci	LMST03020016	Sterol Lipids [ST]	Secosteroids [ST03]	Vitamin D3 and derivatives [ST0302]
**Metabolite Feature Name**	**HMDB**	**Kegg Compund ID**		
2-Methylbutyrylglycine	HMDB00339			
Cystathionine	HMDB00099	C02291		
N-acetyl-Cysteine	HMDB01890	C06809		

## Discussion

Advances in mass spectrometry technology have enabled the detection and quantification of a large number of metabolites in a complex biological sample and provided the opportunity to gain insights into fundamental biological processes by system level analysis [40]. Metabolic profiling is also discussed to have a high potential in the discovery of biomarkers and several studies have applied metabolic profiling successfully, to gain new insights into cell biology, physiology and disease development, especially in neuroscience where it is also discussed as a laboratory tool for diagnosis [41,42]. In the present study for the first time an untargeted comprehensive metabolite profiling of the prefrontal cortex and serum of cattle with distinct temperament types was performed to further explore the molecular divergence between temperament types in cattle and to identify new targets for further research of cattle temperament. First insights indicated that a differentiation between temperament types is possible based on the abundance of prefrontal cortex and serum metabolites in response to the slaughter procedure and that the differences are attributed to metabolites that are related to C21 steroid metabolism and oxidative stress which are known to be involved in the stress and fear response.

### Prefrontal cortex metabolites with high relevance in the classification of temperament types

The prefrontal cortex is discussed to play a central role in cognition control comprising goal or context representation, attention allocation, problem solving, decision making and behavioral planning [43]. Additionally, several studies have shown the importance of the prefrontal cortex in fear and anxiety disorders [44] as well as in the regulation of the glucocorticoid stress response and the fear response mediated by the amygdala [45]. In farm animals it has been repeatedly reported that pre-slaughter conditions and the slaughter procedure itself provoke a stress response [21,46–48] and the novel environment, unknown humans, the separation from conspecifics as well as the handling and restraining of the animals during the slaughter procedure are assumed to be psychological stressors that can trigger a fear response [9]. In addition, the prefrontal cortex was taken after the animal was stunned and exsanguinated in contrast to the serum samples, indicating that the metabolic profiles of the prefrontal cortex were not only affected by psychological but also by physical stress. However, studies in farm animals indicate that the physiological response to the pre-slaughter conditions is still eminent after exsanguination to some extend [46,48,49] and all animals were exposed to the same stressors. Therefore differences between temperament types in metabolite profiles of the prefrontal cortex after slaughter could provide additional information about molecular pathways differentially regulated in the different temperament types, although it remains unclear whether these differences are attributed to differences in the fear or stress responsiveness or the response to physical stress of the different temperament types. PCA of the complete prefrontal cortex metabolite data obtained at an age of 1341 ± 98 days and plotting of the PC-scores of the first two PCs showed a moderate discrimination at least between the distinct temperament types (Fig 1A). This indicated that there are underlying metabolic differences between the temperament types. Behavioral differences and related endocrinological measurements have been shown in other studies to be stable over time to some extend [3,50,51]. SPLS-DA and the Wilcoxon-Mann-Whitney-Test further revealed that the observed differences were predominantly attributed to significant differences in the abundance of glycerophopholipids, fatty acyls, and sterol lipids. Most conspicuous was the identification of sterol lipid 5a-tetrahydrocorticosterone as a metabolite with a high relevance in the classification of temperament types (Table 1) and the observed significant higher abundance in the fearful/neophobic-alert animals in comparison to all other temperament types (Table A in S3 File, Fig 3A). 5a-tetrahydrocorticosterone is a 5alpha-reduced metabolite of corticosterone that exhibits glucocorticoid activity via glucocorticoid receptor binding and activation [52,53]. Additionally, it has been shown to be a positive allosteric modulator of the γ-aminobutyric acid receptor, a receptor for the major inhibiting neurotransmitter γ-aminobutyric acid (GABA) that tends to decrease neuronal excitability upon activation [54]. Increased levels of 5a-tetrahydrocorticosterone have been found in rats after acute stress and a classical negative feedback effect on the hypothalamic-pituitary-adrenal axis as well as an anti-inflammatory effect of 5a-tetrahydrocorticosterone have been reported [52,54,55]. In contrast, intense or prolonged stress resulting in a sustained release of glucocorticoids is also known to be involved in stress induced atrophy in the hippocampus and other brain regions as well as to promote neuronal damage [56,57]. Therefore the differences in the abundance of 5a-tetrahydrocorticosterone were the first indication of substantial differences in the stress response to the slaughter procedure at least between the fearful/neophobic-alert and all other temperament types. In addition, the observed significant higher abundance of glucocorticoid 5a-tetrahydrocorticosterone in the fearful/neophobic-alert animals is concordant to the significant higher abundance of intermediates of gluconeogenesis [53] like glucose, glucose-6-phophat and fructose-6-phosphat (Table A in S2 File) at least in comparison to the indistinct and subdued/uninterested-calm cows, because glucocorticoids are known to stimulate gluconeogenesis [58]. Differences related to energy metabolism have also been reported in studies investigating the impact of temperament on meat quality [46,48,49] or in response to immune challenge [59]. As previously mentioned, stress has also the potential to damage neurons if feedback mechanisms fail to shut down the stress response or in cases of acute, prolonged or repeated stress [57]. Oxidative stress that is induced by the generation of free radicals after the release of excitatory amino acids and the activation of the second messenger system within the stress response, is discussed to be a main mechanism causing neuronal damage [60]. Antioxidant vitamins have been shown to reduce oxidative stress in the rat brain in response to stress induced by a restraint test [61] and [13] have shown that mice selected for high anxiety-related behavior showed a lower total antioxidant capacity in comparison to mice selected for low-anxiety related behavior. In this study we could observe significant differences in the abundance of vitamins (Table 1, Table A in S3 File) that are discussed to be involved in decreasing oxidative stress by different mechanism like ascorbate [61], vitamin K1 hydroquinone [62] and vitamin D3 and derivatives [63]. These differences could therefore indicate differences in the stress response to the slaughter procedure resulting in differing levels of oxidative stress or differing mechanisms used by the different temperament types to cope with or tolerate oxidative stress. Differing levels of oxidative stress were further indicated by significant differences in the abundance of the fatty acyl N-(2-hydroxyethyl)icosanamide (Table 1, Table A in S3 File). N-acylethanolamines like N-(2-hydroxyethyl)icosanamide are involved in the regulation of the inflammatory immune response and are discussed to protect from neuronal death [64]. Several studies have shown an increase in N-acylethanolamines after ischemia and excitotoxicity, and the increase of N-acylethanolamines is suggested to be a defense mechanism against NMDA-receptor mediated excitotoxicity [65,66]. Similar, the differences in the abundance of glycerophospholipids could reflect further mechanisms differentially regulated between temperament types in the allostatic response triggered by the slaughter procedure. Glycerophospholipids have diverse functions in neuronal membranes. They are important for the maintenance of the structure and function of membranes and serve as precursors for important second messengers that are generated, for example, in the response to the release of excitatory amino acids within the stress response, and they are also discussed to be involved in the regulation of enzyme activities, apoptosis, and to protect against oxidative stress [67,68].

To further elucidate differences in the response to the slaughter procedure, a detailed view on the abundance of the main excitatory neurotransmitters glutamate and aspartate [69] as well as on the main inhibitory neurotransmitter GABA [70] was performed. Although most neurotransmitters had no high relevance in the classification of temperament types, significant differences between temperament types could be observed (Table 4, Table A in S2 File). Most notably was the highest abundance of GABA in the subdued/uninterested-calm and inconsistent animals as well as the overall low abundance of all identified neurotransmitters in the fearful/neophobic-alert temperament type. GABA has been shown to have anxiolytic and sedative effects, to be involved in the acquisition and extinction of fear memory and decreased levels of GABA have been found in several mood and anxiety disorders [44,71,72]. The high abundance of GABA and the lower abundance of glucocorticoid 5a-tetrahydrocorticosterone in the subdued/uninterested-calm animals in contrast to the fearful/neophobic-alert temperament type suggested that fear or the emotional evaluation of the thread emanating from the slaughter procedure is an important factor triggering different metabolic responses in dependence of the temperament type. This would be in agreement to behavioral studies in cattle suggesting fear as a major psychological factor of cattle behavior [20,23]. Interestingly, a decreased concentration of GABA but also of glutamate and aspartate have been reported in the prefrontal cortex and plasma of humans suffering from a melancholic major depressive disorder [73], and early life stress in animal models, has been reported to cause decreased glutamate and glutamine levels, increased corticosterone levels in adulthood [74], and to enhance adult anxiety [75]. This indicates that very early life experiences could have had a large impact on the development of the different temperament types in our study, which is most evident in the fearful/neophobic-alert animals due to the generally high abundance of most vitamins, N-acylethanolamines and glycerophopholipids in addition to the highest abundance of glucocorticoid 5a-tetrahydrocorticosterone and the lowest abundance of most neurotransmitters in comparison to the other temperament types.

**Table 4:** Significant differences between temperament types in the abundance of selected excitatory and inhibitory neurotransmitters in the prefrontal cortex.

Temperament type	Glutamic acid		Aspartic acid		GABA (4-amino-Butanoic acid)
fearful/neophobic-alert	↓		↓		↓
interested-stressed		↓		↓	
outgoing/neophilic-alert	↑	↑	↑		
uninterested-calm	↑		↑	↑	↑
indistinct	↑				↑

Significant differences (*p* < 0.05) between temperament types are indicated by arrows.

Further differences observed between temperament types in the prefrontal cortex are difficult to discuss due to the lack of information about the specific function of some metabolites. 2,4-dihydroxy-Butanoic acid for example had a high relevance in the classification of temperament types and showed significant differences between temperament types (Table 1, Table A in S3 File). 2,4-dihydroxy-butanoic acid has been identified as a potential predictive biomarker in Alzheimer’s disease [76] and increased amounts have been observed in the urine in cases of succinic semialdehyde dehydrogenase deficiency [77]. Further knowledge about a distinct function or the metabolism of 2,4-dihydroxy-butanoic is still lacking.

### Serum metabolites with high relevance in the classification of temperament types

A prerequisite for a metabolic marker that is thought to contribute to the classification of temperament types in cattle is the accessibility of the tissue. Blood samples can be taken minimally invasive during routine veterinary inspections and are well suited for larger scaled studies. Blood is a highly informative sample type that is involved in transport and communication between organs and tissues and is often used to gain additional information about endocrinological parameters in behavior studies. In our study, serum metabolites showed no obvious discrimination between temperament types in the PCA of the complete serum data in contrast to the prefrontal cortex (Fig 1), and substantial differences between the serum and prefrontal cortex metabolite profiles were observed. These differences could arise from the different biophysical characteristics of the tissues that affect the detection of metabolites, the blood brain barrier hindering a large proportion of metabolites to freely circulate, the different functions of the tissues themselves, and the different time-points the samples were collected (before stunning and post mortem). However, the sPLS-DA could identify 51 serum metabolite features to have a high relevance in the classification of temperament types and significant differences between temperament types could be observed in the Wilcoxon-Mann-Whitney-Test (Table 1, Table B in S3 File). Most conspicuous was the identification of pregnenolone and allopregnanolone as well as of cysthationine and N-acety-cystheine as metabolites with a high relevance in the classification of temperament types (Table 1). Pregnenolone and allopregnanolone are C21 steroids and pregnenolone is an important metabolite in the metabolism of allopregnanolone and other adrenal corticosteroids, because the conversion of cholesterol into pregnenolone is a rate limiting step in the biosynthesis of steroids. Both pregnenolone and allopregnanolone are discussed to have antidepressant-like effects and reduce anxiety via GABA-receptor modulation, and reduced or increased levels of allopregnanolon have been reported to be associated with major depression, impulsive aggression and other anxiety related disorders, respectively [78–80]. In addition, [41] reported that pregnenolone in combination with other steroids could assist in the diagnosis of schizophrenia as predictive biomarker and [78] showed that administration of pregnenolone resulted in increased allopregnanolone levels and enhanced the activity of brain regions linked to the regulatory control over emotion as well as to the connectivity between the dorsal medial prefrontal cortex and amygdala [81], both important brain regions in the regulation of the fear and stress response. Allopregnanolone levels were also significantly correlated with low-anxiety and exploratory behaviors in rats [78], which is in agreement to the behavior of the outgoing/neophilic-alert and interested-stressed animals that showed the highest abundance of allopregnanolone (Fig 3B) and had more often contact to or longer contact with the novel object or human.

Similar to allopregnanolone, N-acetyl-cysteine has been shown to decrease immobility time in a forced swimming test in rats, indicating an antidepressant like effect [82,83]. In contrast to allopregnanolone that showed a dose dependent decrease in the immobility time in rats, N-acetyl-cysteine showed a dose dependent but U-shaped decrease in immobility time and [83] additionally indicated that high doses might also decrease exploratory behavior in an open arena. The effects of allopregnanolone are discussed to be maintained by the modulation of the gabaergic system [82], whereas the effects of N-acetyl-cysteine were related to the function of N-acetyl-cysteine as a reactive oxygen species scavenger preventing stress-induced neuronal damages [83]. This further supports previous findings in the prefrontal cortex indicating differing oxidative stress levels or differing mechanisms used to cope with or tolerate oxidative stress.

Another metabolite with high relevance in the classification of temperament types that is related to oxidative stress was cystathionine. Cystathionine is a metabolite in the transsulfuration pathway that converts homocysteine to cysteine, the limiting educt in the synthesis of glutathione which is a major antioxidant [84]. Cystathionine is discussed to play an important role in neurodegenerative diseases like Parkinson's disease and Alzheimer's disease [85], and in autism, decreased levels of cystathionine have been reported that are discussed to be related to an increased vulnerability to oxidative stress [86]. In addition, cystathionine γ-lyase has been shown to protect against oxidative stress in a striatal cell line Huntington’s disease model [87]. Interestingly, homocysteine another metabolite in the transsulfuration pathway showed a significantly higher abundance in the indistinct and subdued/uninterested-calm temperament types in comparison to the fearful/neophobic-alert animals in the prefrontal cortex.

## Conclusions

Untargeted metabolite profiling enabled the detection of a large number of metabolite features in the prefrontal cortex and serum of animals with distinct temperament types and provided insights into molecular mechanisms related to differences in the response to the slaughter procedure of different temperament types. Differences in the abundance of metabolites related to C21 steroid metabolism, like 5a-tetrahydrocorticosterone, pregnenolone and allopregnanolone, and oxidative stress, like cystathionine, N-acetyl-cysteine and ascorbate, between the four temperament types indicated that molecular pathways involved in the stress and fear response are regulated temperament type dependent in the response to the slaughter procedure. Whether these differences are due to a genetic predisposition or due to the different experiences of the animals needs further research. Particularly the serum metabolites pregnenolone and allopregnanolone are interesting targets for further research of cattle temperament due to the discussed anxiolytic effects and the effects on the activity of brain regions linked to the regulatory control of emotion. Nevertheless, it has to be considered that this study was an exploratory approach to identify new targets for further research of cattle temperament conducted in a relatively small but homogeneous group of cows in regard to age and reproductive status, which both could affect the abundance of metabolites as it has been shown for allopregnanolone [78,88].

## Supporting Information

S1 File Principal component loadings of the measured behaviours. Loadings of the behaviors in principal component (PC) 1 and PC2 gained from the principal component analysis of the novel object and novel human test. Loadings for the novel object test are from [26].
(XLS)

S2 File List of serum and prefrontal cortex metabolite features. Metabolite feature names, measuring technique and *p-*values for the Kruskal-Wallis- and the Wilcoxon-Mann-Whitney-Test are provided for prefrontal cortex (Table A) and serum (Table B).
(XLS)

S3 File Summary results for serum and prefrontal cortex. Metabolite feature names, measuring technique, mean VIP-score and the number of occurrence in the outer cross validation runs as well as *p-*values for the Kruskal-Wallis- and the Wilcoxon-Mann-Whitney-Test are provided for prefrontal cortex (Table A) and serum (Table B).
(XLS)

S4 File Prefrontal cortex data. Log transformed, mean-centered and unit variance scaled prefrontal cortex metabolite data.
(TXT)

S5 File Serum data. Log transformed, mean-centered and unit variance scaled prefrontal cortex metabolite data.
(TXT)
